# Joint Destruction Is Associated With All Types of Cardiovascular Events in French Rheumatoid Patients: A Real-Life Study With Very Long Follow-Up

**DOI:** 10.3389/fmed.2020.556086

**Published:** 2020-10-30

**Authors:** Marie Robert, Arnaud Hot, François Mifsud, Ndiémé Ndongo-Thiam, Pierre Miossec

**Affiliations:** ^1^Department of Clinical Immunology and Rheumatology, Immunogenomics and Inflammation Research Unit EA 4130, University of Lyon 1, Hôpital Edouard Herriot, Lyon, France; ^2^Department of Internal Medicine, Immunogenomics and Inflammation Research Unit EA 4130, University of Lyon 1, Hôpital Edouard Herriot, Lyon, France

**Keywords:** rheumatoid arthritis, joint destruction, inflammation, cardiovascular diseases, vessels

## Abstract

**Objective:** Rheumatoid arthritis (RA) leads not only to joint destruction but also to systemic manifestations, with an increased incidence of cardiovascular events (CVE). Many studies have shown a link between RA severity and CV risk, but the duration of follow-up remains often insufficient to allow a conclusion. The CVE definition was generally reduced to myocardial infarction and stroke, and few studies were conducted in non-Anglo-Saxon countries with low CV incidence. This study aimed to assess the relationship between joint destruction and the occurrence of different types of CVE in a large cohort of French RA patients with a long-term follow-up.

**Methods:** This historical cohort study included 571 RA patients followed between 1992 and 2012 in Lyon, France. The primary endpoint was the first occurrence of a CVE. Logistic regressions were used to identify factors associated with CVE occurrence. Cox proportional hazard models were performed as a separate analysis to take advantage of the long-term follow-up.

**Results:** During a mean follow-up of 16.1 years, 30.3% of patients experienced a CVE, mostly acute arterial events. Joint destruction was associated with an increased risk of CVE [odds ratio = 3.72; 95% confidence interval (CI), 1.09–15.35; *p* = 0.047] among non-smoker RA patients. A survival analysis revealed that joint destruction was associated with a shorter time to onset of the first CVE only among non-smokers (hazard ratio = 3.44; 95% CI, 1.07–11.04; *p* = 0.038).

**Conclusion:** Joint destruction is associated with CVE occurrence in RA patients from a population with a lower incidence of CV disease. This study suggests that RA patients, especially those with destruction, merit the institution of precise guidelines to manage this CV risk, and trials are required to evaluate them.

## Introduction

Rheumatoid arthritis (RA) is a chronic inflammatory disease characterized by joint destruction (1). The effects of RA on life expectancy were rather recently identified, with a crucial impact of RA on the risk of cardiovascular events (CVE). A detailed analysis showed the modest contribution of classical CV risk factors, suggesting a direct link between CVE occurrence and chronic inflammation (2–4). Although the exact cause of RA remains unknown, the contribution of pro-inflammatory cytokines is now well-established. For instance, tumor necrosis factor-alpha (TNFα), interleukin (IL)-6, and IL-17 have a direct contribution to joint damage and to the development of accelerated atherosclerosis and premature CVE (4–9). The control of inflammation with inhibitors of these cytokines appears to reduce CV risk in RA, and more trials are ongoing (4, 10, 11).

The prevalence of CVE in the general population and in RA patients varies a lot between Anglo-Saxon and non-Anglo-Saxon countries (12, 13). Even if the mode of living and diet should contribute to the difference (14, 15), the impact of RA-related risk factors remains to be clarified. Among them, an association between the occurrence of CVE and joint destruction has been suggested in a case–control study but was not confirmed after adjusting for confounding factors (16, 17). Another important factor is the duration of follow-up to reach a definite conclusion, with very few studies with a 15-year follow-up. Moreover, the definition of CVE is generally reduced to acute arterial events, with a focus on myocardial infarction and stroke (12, 16, 17).

This study aimed to analyze the link between joint destruction and the occurrence of different types of CVE in a large non-Anglo-Saxon French RA population with over 15-year follow-up.

## Materials and Methods

### Patients and Study Design

A historical cohort study was designed using 1,194 RA patients followed between 1992 and 2012 at the Clinical Immunology and Rheumatology Department in a tertiary university hospital in Lyon (Hôpital Edouard Herriot), France. Demographic and clinical data and RA-related parameters were collected during the whole follow-up for each patient and recorded in a computer database. The inclusion criteria were that the patients had to fulfill the American College of Rheumatology criteria for RA diagnosis (18), with available clinical and biological follow-up data collected between 1992 and 2012. In 2012, CVE status was available in 698 cases out of 1,194, and these patients were included in the study. Then, patients were excluded when a CVE preceded RA diagnosis or when follow-up duration was <3 years; 571 patients were finally included. The cutoff of 3 years was chosen because data from literature and clinical experience have concluded that most of RA destruction occurs in this period of time, called the window of opportunity (19). So far, there is no similar equivalent for the effect of inflammation on CV risk, so the same cutoff was selected. The method used to select the patients included in the study may have induced some bias as only patients with information on the occurrence of CVE were selected. Considering the objective of the study, this method although imperfect, appeared to be the most appropriate one. All patients signed an informed consent. The study complied with the local ethics committee and was approved by the Ministry of Research (reference number: AC-2010-1164).

### Outcome Measurements

The primary outcome was the first occurrence of a CVE defined as: myocardial infarction, stroke, acute limb ischemia, chronic heart failure, cardiomyopathy or atrial fibrillation, deep vein thrombosis, and pulmonary embolism. Data regarding CVE were retrospectively obtained from the hospital chart by calling the general practitioner or calling the patient. Both fatal and non-fatal CVE were considered.

The patients were assigned to two groups according to the presence or the absence of radiographic wrist bone destruction, defined by the Larsen score that ranges from 0 to 5. RA with scores equal to 0 or 1 were considered as non-destructive, whereas scores superior or equal to 2 were considered as destructive. The Larsen score assigned to each patient was the highest observed during the whole follow-up. RA biomarkers (anti-citrullinated protein antibodies, ACPA, and rheumatoid factor, RF) were also collected, and disease activity was reported with the erythrocyte sedimentation rate (ESR) and disease activity score-28 (DAS-28). Median DAS-28 corresponds to the median of all values available during the follow-up. DAS-28 value at diagnosis was not reported as this marker was not routinely used at the beginning of the study. All the treatments for RA used during the whole follow-up were collected. Classical CV risk factors were also recorded. All exposition variables were collected during the whole follow-up. The patients were defined as smokers if they had ever smoked during the follow-up.

### Statistical Analysis

The main goal of the study was to assess the link between RA radiological severity (Larsen score) and the occurrence of CVE in 571 RA patients. Regarding the retrospective collection of CVE, data were first analyzed as a case–control study. A logistic regression model was used to test the association between joint destruction and other factors with the occurrence of CVE. Odds ratios (OR) were calculated in a univariate analysis. Then, a multivariate logistic regression was performed to calculate the same OR adjusted to all the variables significantly associated with the risk of CVE in the univariate analysis and to assess the interactions between these variables. All logistic regressions and tests were performed using R software with the glm function (20). As logistic regression analysis cannot account for differences in disease and follow-up duration, a survival analysis was performed to confirm the results in a framework able to properly handle right-censoring. In a second analysis, survival analysis was used to estimate the association between Larsen score and time to onset of the first CVE during patient's follow-up. Cox proportional hazard regression model was adjusted to the same covariates with the coxph function of the R library survival (21, 22). Coefficients were tested at a significance level of 0.05. The proportional hazard assumption was tested with chi-square test using the cox.zph function (21, 22). No imputation was performed for missing data, and incomplete cases were not considered when computing the OR or the hazard ratio (HR) by regression. Multivariate logistic regression and Cox model were trained on the 379 patients, for which all considered exposition variables were available.

## Results

### Characteristics of Patients

The study population included 571 RA patients, and the mean (±standard deviation) duration of follow-up was 16.1 (±9.9, min: 3–max: 58) years; 73.4% were female (419/571), and the mean age at diagnosis was 47.2 (±14.1) years. The average body mass index (BMI) was 25.7 (±5.2) kg/m^2^; 39.8% (218/548) of RA patients smoked. High blood pressure was observed in 45.0% (243/540) of patients, diabetes in 11.2% (60/534), and dyslipidemia in 34.4% (176/512). Regarding RA treatments, most patients were on methotrexate (538/567, 94.9%) and received steroids (426/567, 75.1%), non-steroidal anti-inflammatory drugs (NSAIDs) (346/522, 66.3%), and biologics (284/571, 49.7%). Considering disease severity, the mean Larsen score was 2.15 (±1.7), and 58.1% (327/563) of the patients were classified as having destructive RA with a Larsen score higher than 2. Regarding disease activity, the median DAS-28 was 3.5 (±1.1), and the mean ESR was 31.5 (±23.9) mm per hour. ACPA were positive in 61.2% (300/490) and RF in 62.2% (334/537) of the patients. These data are summarized in Table 1. The characteristics of patients are also presented according to their CVE status in Supplementary Table 1.

**Table 1:** Population characteristics at the time of analysis.

**Population description (*****N*** **=** **571 patients)**		
**Variables**	**Values**	**Missing data**
**Global characteristics**		
Male sex—no. (%)	152 (26.6%)	
Year of birth	1947 ± 13.3	
Age at diagnosis—years	47.2 ± 14.1	
BMI—kg/m^2^	25.7 ± 5.2	48 (8.4%)
CVE—no. (%)	173 (30.3%)	
Myocardial infarction	50 (8.8%)	
Stroke	28 (4.9%)	
Acute limb ischemia	23 (4.0%)	
Chronic heart failure	14 (2.5%)	
Cardiomyopathy	25 (4.4%)	
Deep vein thrombosis	27 (4.7%)	
Pulmonary embolism	6 (1.1%)	
Death—no. (%)	60 (11.3%)	38 (6.7%)
Myocardial infarction	15 (2.8%)	
Stroke	6 (1.1%)	
Other CVE	7 (1.3%)	
Infection	15 (2.8%)	
Cancer	10 (1.9%)	
Other	6 (1.1%)	
Length of follow-up—years	16.1 ± 9.9	
**RA characteristics**		
ACPA positivity—no. (%)	300 (61.2%)	81 (14.2%)
RF positivity—no. (%)	334 (62.2%)	34 (6.0%)
Larsen score	2.15 ± 1.7	8 (1.4%)
Larsen score <2—no. (%)	236 (41.9%)	
Larsen score ≥2—no. (%)	327 (58.1%)	
Median DAS-28	3.5 ± 1.1	143 (25.0%)
ESR—mm/h	31.5 ± 23.9	51 (8.9%)
**CV risk factors**		
Never smoked—no. (%)	330 (60.2%)	23 (4.0%)
High blood pressure—no. (%)	243 (45.0%)	31 (5.4%)
Diabetes—no. (%)	60 (11.2%)	37 (6.5%)
Dyslipidemia—no. (%)	176 (34.4%)	59 (10.3%)
**Treatments**		
NSAIDs—no. (%)	346 (66.3%)	49 (8.6%)
Steroids—no. (%)	426 (75.1%)	4 (0.70%)
Methotrexate—no. (%)	538 (94.9%)	4 (0.70%)
Biologics—no. (%)	284 (49.7%)	
Immunosuppressive agents—no. (%)	76 (13.3%)	
DMARDs—no. (%)	267 (46.7%)	

*If a patient had more than one cardiovascular event, the first one was used for the analysis. Quantitative variables are reported as mean ± standard deviation*.

*RA, rheumatoid arthritis; CV, cardiovascular; CVE, CV event(s); BMI, body mass index; ACPA, anti-citrullinated protein antibodies; RF, rheumatoid factor; DAS-28, disease activity score-28; ESR, erythrocyte sedimentation rate; NSAIDs, non-steroidal anti-inflammatory drugs; DMARDs, disease-modifying anti-rheumatic drugs*.

### Occurrence of CVE in RA Patients and Causes of Death

Thirty percent (173/571, 30.3%) of RA patients had at least one CVE during the follow-up period. The most frequent type of CVE was an acute arterial event (101/571, 17.7%), with a majority of myocardial infarction (50/571, 8.8%), then stroke (28/571, 4.9%), and acute limb ischemia (23/571, 4.0%). Heart conditions included chronic heart failure (14/571, 2.5%) and cardiomyopathy (25/571, 4.4%). Venous thrombotic events (VTE) affected 33/571 (5.8%) patients, with a majority of deep vein thrombosis (27/571, 4.7%) (Table 1).

During the whole follow-up period, 11.3% of the patients died (60/533). The main causes of death were myocardial infarction and infection (15/533 for each, 2.8%), followed by cancer (10/533, 1.9%). The overall CV mortality was 5.3% (28/533) (see Table 1 for details).

### CV Risk Factors and Disease Activity Confer a Higher Risk of CVE

**Figure 1:**
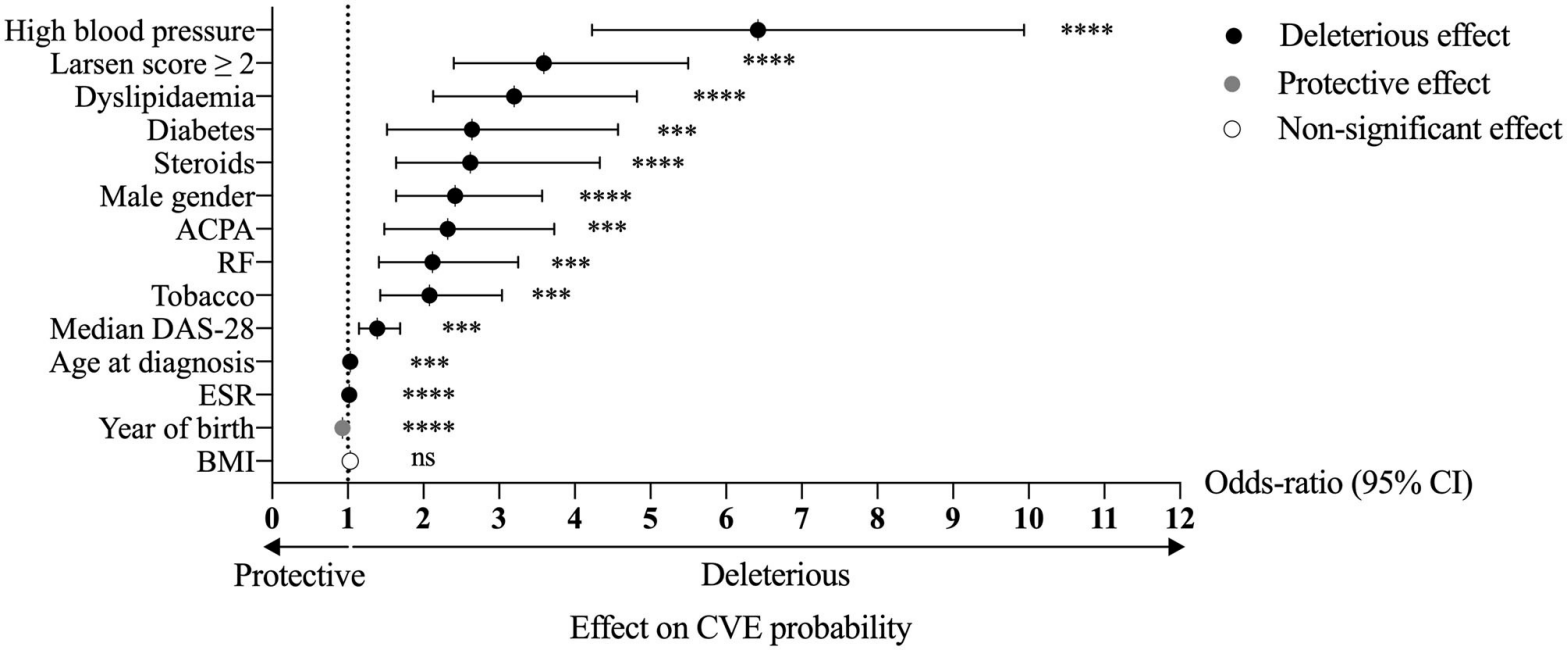
Univariate analysis of the association between the studied variables and the risk of CVE in RA patients. ****p < 0.0001, ***p < 0.001; ns, non-significant; RA, rheumatoid arthritis; CVE, cardiovascular event(s); ACPA, anti-citrullinated protein antibodies; RF, rheumatoid factor; DAS-28, disease activity score-28; ESR, erythrocyte sedimentation rate; BMI, body mass index; CI, confidence interval.

Univariate analysis was performed to assess the association between each variable and the occurrence of CVE (Figure 1). The traditional CV risk factors were all significantly associated with the risk of CVE, except for BMI. High blood pressure was the highest risk factor [*OR* = 6.42; 95% confidence interval (CI), 4.23–9.94; *p* < 0.0001]. The parameters related to disease activity, including ESR and median DAS-28, were also associated with the risk of CVE (ESR: *OR* = 1.02; 95% CI, 1.01–1.03; *p* < 0.0001; median DAS-28: *OR* = 1.39; 95% CI, 1.15–1.69; *p* = 0.00064). Patients with destructive RA (Larsen score from 2 to 5) had a higher risk of CVE than those without (Larsen 0–1), with a univariate OR of 3.59 (95% CI, 2.40–5.50; *p* < 0.0001). ACPA and RF positivity were associated with a higher risk (*OR* = 2.32; 95% CI, 1.48–3.73; *p* = 0.00034 and *OR* = 2.12; 95% CI, 1.41–3.25; *p* = 0.00040, respectively). Steroid use was also associated with a higher risk of CVE (*OR* = 2.62; 95% CI, 1.64–4.33; *p* < 0.0001). No significant associations were found between biologics, disease-modifying anti-rheumatic drugs (DMARDs), and the occurrence of CVE (data not shown).

### Effect of Joint Destruction on the Risk of CVE

Multivariate logistic regression was performed to adjust for the effect of the Larsen score on variables that were independently associated with the risk of CVE (Table 2). An interaction between the effects of the Larsen score and smoking was found. Therefore, the analysis was repeated separately among non-smokers and smokers. The Larsen score was significantly associated with a higher risk of CVE among non-smokers (*OR* = 3.72; 95% CI, 1.09–15.35; *p* = 0.047), but not among smokers (*OR* = 1.33; 95% CI, 0.50–3.55; *p* = 0.57). The global effect of the Larsen score on the whole cohort was at the limit of significance (*OR* = 1.98; 95% CI, 0.97–4.13; *p* = 0.064). However, in a model in which the interaction term between the Larsen score and smoking was included, the effect of the Larsen score for the whole cohort was significant (*OR* = 4.60; 95% CI, 1.46–17.91; *p* = 0.015; data not shown). When the definition of CVE was restricted to acute arterial events (myocardial infarction, stroke, and acute limb ischemia), the effect of the Larsen score on the whole cohort was also significant (*OR* = 6.078; 95% CI, 1.47–41.86; *p* = 0.027).

**Table 2:** Multivariate analysis of the association between the studied variables and the risk of CVE in RA patients.

**Variables**	**Global (whole cohort)**		**Non-smokers**		**Smokers**	
	**Odds ratio (95% CI)**	***P*-value**	**Odds ratio (95% CI)**	***P*-value**	**Odds ratio (95% CI)**	***P*-value**
High blood pressure	4.73 (2.47–9.36)	<0.0001	2.70 (0.99–7.68)	0.055	7.96 (3.26–20.83)	<0.0001
Tobacco	3.50 (1.78–7.06)	0.00034	–	–	–	–
Male gender	2.54 (1.26–5.19)	0.0097	7.31 (1.79–31.43)	0.0057	2.03 (0.83–5.13)	0.12
Median DAS-28	1.54 (1.16–2.06)	0.0031	1.42 (0.93–2.22)	0.11	1.61 (1.07–2.47)	0.024
Age at diagnosis	0.97 (0.94–1.00)	0.043	0.96 (0.92–1.00)	0.064	0.98 (0.93–1.04)	0.53
Year of birth	0.93 (0.89–0.98)	0.0027	0.88 (0.82–0.94)	0.00056	0.98 (0.91–1.04)	0.48
Larsen score ≥2	1.98 (0.97–4.13)	0.064	3.72 (1.09–15.35)	0.047	1.33 (0.50–3.55)	0.57
RF	1.50 (0.69–3.30)	0.31	1.98 (0.54–7.71)	0.31	1.27 (0.43–3.79)	0.66
Dyslipidemia	1.47 (0.78–2.73)	0.23	1.07 (0.37–3.07)	0.90	2.28 (0.97–5.36)	0.057
Steroids	1.24 (0.56–2.90)	0.61	1.24 (0.31–6.72)	0.78	1.43 (0.49–4.34)	0.51
Diabetes	1.11 (0.47–2.56)	0.82	1.53 (0.36–5.78)	0.54	1.01 (0.31–3.23)	0.99
ACPA	0.81 (0.37–1.80)	0.60	0.89 (0.25–3.35)	0.86	0.78 (0.25–2.39)	0.66

*RA, rheumatoid arthritis; CVE, cardiovascular event(s); DAS-28, disease activity score-28; RF, rheumatoid factor; ACPA, anti-citrullinated protein antibodies; CI, confidence interval*.

### Effect of Joint Destruction on the Time to the First CVE During the Whole Follow-Up

**Figure 2:**
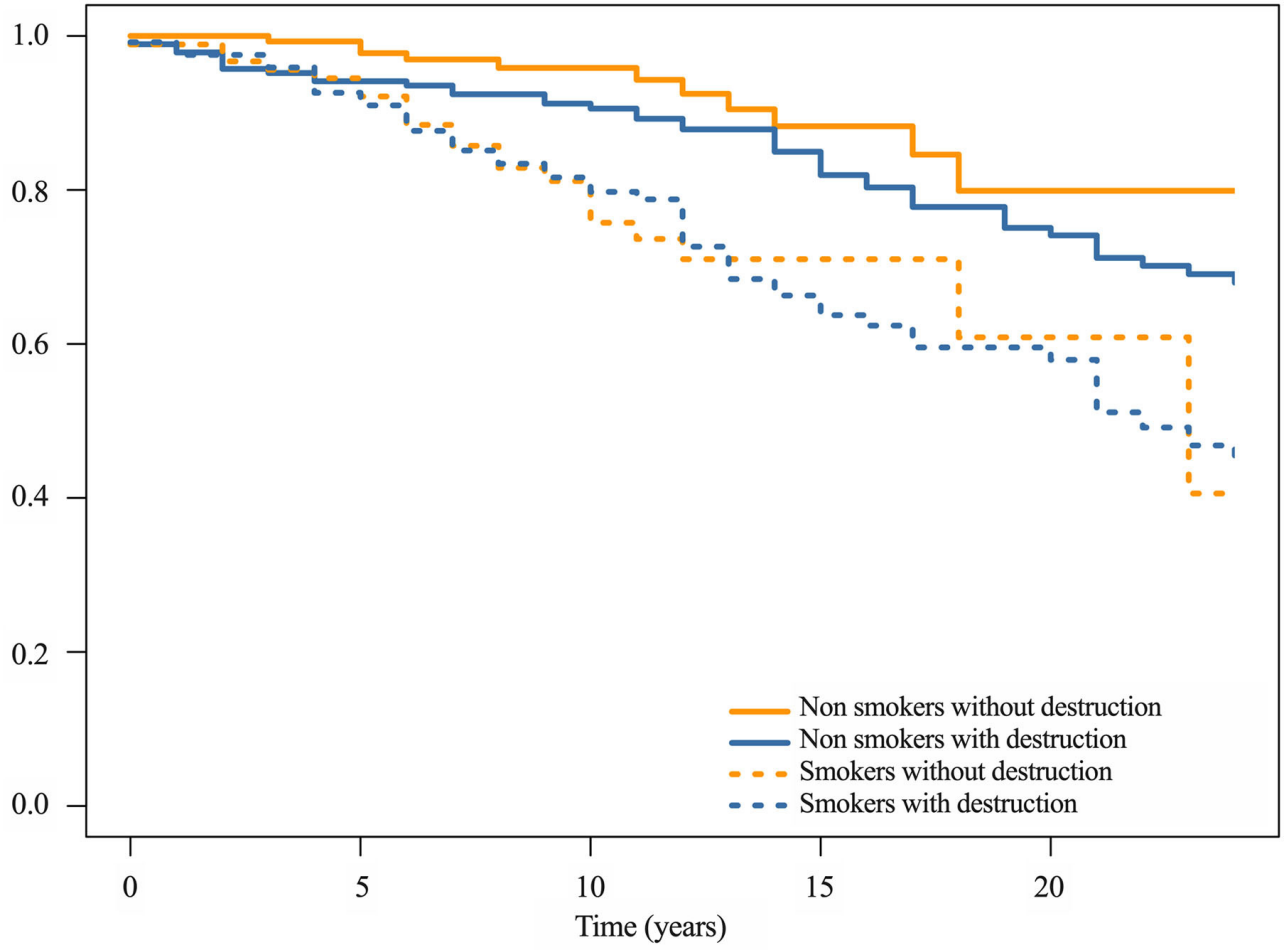
Kaplan–Meier curve of CVE-free survival. If a patient had more than one CVE, the first one was used for the analysis. The patients were defined as smokers if they were currently smoking or had already smoked. The patients were assigned to two groups according to the presence or the absence of radiographic wrist bone destruction, defined by the Larsen score that ranges from 0 to 5. RA with scores equal to 0 or 1 were considered as non-destructive, whereas scores superior or equal to 2 were considered as destructive. RA, rheumatoid arthritis; CVE, cardiovascular event(s).

To take advantage of the longitudinal data available, a survival analysis was performed to investigate the link between Larsen score and time to onset of the first CVE from the beginning of the follow-up in the Rheumatology Department (Table 3). A Cox regression model was used to adjust for the same covariates than in the logistic regression. The effect of destruction on the whole cohort was not significant (*HR* = 1.65; 95% CI, 0.92–2.94; *p* = 0.092). For the non-smokers with a Larsen score ≥2, the time to onset of the first CVE was significantly shorter (*HR* = 3.44; 95% CI, 1.07–11.04; *p* = 0.038), but not for smokers (*HR* = 1.10; 95% CI, 0.54–2.25; *p* = 0.79). Kaplan–Meier curve of CVE-free survival by group is shown in Figure 2.

**Table 3:** Cox regression on time to onset of the first CVE in RA patients.

**Variables**	**Global (whole cohort)**		**Non-smokers**		**Smokers**	
	**Hazard ratio (95% CI)**	***P*-value**	**Hazard ratio (95% CI)**	***P*-value**	**Hazard ratio (95% CI)**	***P*-value**
High blood pressure	3.59 (2.03–6.37)	<0.0001	1.95 (0.80–4.74)	0.14	5.59 (2.58–12.10)	<0.0001
Tobacco	2.37 (1.42–3.94)	0.00096	–	–	–	–
Male gender	1.65 (1.01–2.70)	0.044	4.64 (1.68–12.83)	0.0031	1.43 (0.81–2.53)	0.22
Median DAS-28	1.22 (1.01–1.48)	0.039	1.25 (0.92–1.71)	0.16	1.28 (0.99–1.65)	0.058
Age at diagnosis	1.15 (1.10–1.20)	<0.0001	1.15 (1.07–1.24)	0.00014	1.15 (1.09–1.22)	<0.0001
Year of birth	1.12 (1.07–1.18)	<0.0001	1.09 (1.00–1.18)	0.060	1.16 (1.08–1.24)	<0.0001
Larsen score ≥2	1.65 (0.92–2.94)	0.092	3.44 (1.07–11.04)	0.038	1.10 (0.54–2.25)	0.79
RF	1.49 (0.83–2.68)	0.18	2.31 (0.77–6.96)	0.14	1.24 (0.58–2.64)	0.58
Dyslipidemia	1.42 (0.89–2.25)	0.14	1.29 (0.55–3.06)	0.56	1.80 (0.97–3.35)	0.064
Steroids	1.22 (0.63–2.36)	0.55	1.17 (0.32–4.32)	0.81	1.34 (0.60–3.01)	0.48
Diabetes	0.90 (0.50–1.62)	0.72	1.42 (0.50–4.02)	0.51	0.75 (0.36–1.58)	0.45
ACPA	0.72 (0.39–1.32)	0.28	0.63 (0.22–1.79)	0.39	0.74 (0.33–1.67)	0.47

*RA, rheumatoid arthritis; CVE, cardiovascular event(s); DAS-28, disease activity score-28; RF, rheumatoid factor; ACPA, anti-citrullinated protein antibodies; CI, confidence interval*.

## Discussion

It is now well-established that RA patients have an increased risk of CVE (23). Even though chronic inflammation remains considered as a CV risk factor, few studies have fully confirmed its role in RA premature mortality, mainly because of insufficient follow-up. In addition, it remains to be demonstrated that the same conclusion applies to patients from a population with a lower risk of CV disease. In this study, the contribution of each RA-related risk factor in the risk of CVE was assessed. CVE definition included not only arterial and cardiac diseases but also VTE. The main finding was that destructive RA was linked to a higher risk of CVE in non-smokers. Survival analysis revealed that destruction was associated with a shorter time to occurrence of the first CVE compared to non-destructive RA in non-smokers.

To assess the link between RA and CVE, data for demographic and RA activity parameters were prospectively collected since 1992. This RA cohort was not different from other RA cohorts regarding sex ratio, age at diagnosis, and ACPA/RF positivity (23, 24). The percentages of smokers and patients with high blood pressure, diabetes, and dyslipidemia were rather higher than in the general French population (25). The multivariate analysis could then adjust for these covariates. This difference could be due to the long-term follow-up and the aging of the study population during the last two decades.

While our non-Anglo-Saxon population is supposed to have a lower CV risk (12–14), this long follow-up study confirmed the high prevalence of CVE in RA patients, with 30.3% (173/571) of the cohort with at least one CVE. Myocardial infarction was the most common CVE and the leading cause of death (26), followed by stroke. These results confirm the major role of RA in accelerated and severe atherosclerosis (4, 27). Although arterial complications were the most common CVE in RA, our CVE definition was not reduced to acute arterial events, unlike most previous studies (12, 16, 17). Indeed RA patients had a high proportion not only of heart disease (28) but also of VTE (5.8%, 33/571) compared to the frequency observed in the French population (29). This reflects the effects of inflammation on coagulation and fibrinolysis.

Traditional CV risk factors alone could not explain the observed increase of CVE occurrence, particularly in Mediterranean countries (2, 3). Regarding the contribution of RA-related factors, the main finding of the univariate analysis was that patients with a destructive RA had a higher risk of CVE than patients without. To assess the effect of destruction in currently smoking patients or past smokers, these patients were separated from non-smokers. Smoking is associated with both RA development and CVE. As tobacco is also associated with RA activity, former, and current smokers were combined considering that all lesions, specifically destruction, acquired during the period of smoking would not regress. Joint destruction was clearly demonstrated as a risk factor for CVE in the non-smokers. When the interaction term between Larsen score and tobacco was considered and the CVE definition was restricted to acute arterial events, joint destruction was significantly associated with CVE occurrence. In addition, to account for differences in disease and follow-up duration, Kaplan–Meier and Cox regression were performed to properly handle right-censoring. A survival analysis revealed that the non-smoking patients had a shorter time to onset of the first CVE when they had destructive RA. For the first time, the link between Larsen score and CVE occurrence was clearly demonstrated especially in non-smoking patients, indicating that its contribution was independent from that of smoking. The dual CV and immunological contribution of smoking was too important to extend these results to the whole population. Nonetheless, ACPA and RF, known to predict joint destruction, were associated with the occurrence of CVE only in the univariate analysis (30). Similar results were found in recent studies, with an association between ACPA/RF positivity and CVE occurrence that was no longer significant after adjusting for all CV risk factors (31). However, ACPA/RF positivity, especially at high levels, is considered as a poor prognosis factor (32). The presence of these markers helps in identifying patients at risk of developing an aggressive disease with uncontrolled inflammation that, in turn, increases the risk of CVE. This may explain why the new EULAR CV risk recommendations have considered ACPA and RF as CV risk factors (33). Finally, the association with the year of birth could be explained by the evolution of RA diagnosis and management. Today, RA diagnosis is made earlier than in the 1990s, allowing rapid and better disease control. The rapid control of RA with all new treatments now available also limits destruction and reduces chronic inflammation that, in turn, reduces CV inflammation and then CVE occurrence. These elements explain the “protective” role of the variable year of birth. The very long follow-up required to study properly the occurrence of CVE in RA induces obvious limitations as care changes over time, affecting diagnosis and follow-up, loss of follow-up, and heterogeneity of drug use. In addition, data from literature and clinical experience revealed that most of RA destruction occurs in the first 2–3 years of the disease, referred to as the window of opportunity (19). Two phenotypes of patients can be identified: the first without joint destruction after 2 years of the disease that will not appear over time and at low risk of CVE and the second phenotype with a rapidly destructive disease that will tend to increase over time and at a high risk of CVE. This is a rather binary situation as there is no real dose effect between the level of destruction and the risk of CVE. This concept remains to be fully applied to CV risk, and EULAR recommendations of CV risk management in RA insist on the need of an optimal and a rapid disease control (33).

Overall, joint destruction level is a major risk factor for CVE in RA, but its contribution is reduced by that of tobacco, diabetes, or high blood pressure. Absence of these classical risk factors leaves RA patients still at a higher risk. Even if it would not be sufficient in RA, the control of traditional factors remains crucial (34). Smoking is a preventable risk factor for both RA and CVE as it could act not only through its classical CV effects but also directly on the disease itself, the induction of ACPA, or the treatment response (30, 35, 36). However, the increased CV mortality in patients without the classical risk factors clearly indicates the need of an optimal control of RA disease. These elements corroborate the EULAR recommendations that emphasize the need not only for control of disease activity but also of traditional CV risk factors (33).

Joint destruction results from a long-term evolution of the disease, while prevention of CV risk in these patients is required since RA diagnosis (37). Therefore, it remains crucial to identify early patients that would develop severe and destructive disease (38), but predictive biomarkers are still lacking. These biomarkers could be pro-inflammatory cytokines that play a role in both the early induction and the late chronic stages of RA. They also act on the CV system and contribute to the premature mortality observed (4). For instance, IL-17 bioactivity is associated with destruction in RA patients and with blood vessel inflammation and thrombosis (8, 39–42). Prospective studies to evaluate the use of such predictive biomarkers of CVE occurrence in RA would be of interest (43). Such studies could contribute to improve scores developed to estimate the risk of a future CVE for an individual patient. For instance, the widely used Framingham Risk Score and the Systematic Coronary Risk Evaluation underestimate the CV risk in patients with inflammatory diseases as they do not take into account the added risk of inflammation (44).

Therefore, RA patients with destruction constitute a particularly vulnerable group for CV risk that supports the institution of precise guidelines to manage this risk (45, 46). Long-term trials would be necessary to evaluate novel therapeutic interventions such as pro-protein convertase subtilisin-kexin type 9 (PCSK9) inhibitors or high-intensity statin therapy (47, 48). Specific RA therapies also lowered the CV risk in these patients as the use of methotrexate was associated with a decreased CV mortality (11, 49). This reduction was not observed in patients with a history of myocardial infarction or multivessel coronary artery disease and either type 2 diabetes or metabolic syndrome without chronic inflammatory disorders (50). Conversely, the use of IL-1ß inhibitor (canakinumab) showed promising results in patients with myocardial infarction and a blood level of C-reactive protein of 2 mg/L or more despite aggressive secondary prevention strategies (51). Therefore, the use of unspecific and specific therapies to manage the CV risk needs to be rapidly considered in the group of RA patients, especially in destructive RA.

In conclusion, the increased CV mortality in patients without the classical risk factors clearly indicates the need of an optimal control of RA disease. This demonstration was made in a non-Anglo-Saxon population where the CV risk is lower, in part because of the lower contribution of the traditional risk factors. However, the high prevalence of CVE in RA patients illustrates that the urgent need to define guidelines to manage this CV risk and trials to evaluate them are necessary. The long-term follow-up over 15 years shows that an early RA diagnosis is required to avoid destruction and prevent CV disease development in these patients.

## Data Availability Statement

The raw data supporting the conclusions of this article will be made available by the authors, without undue reservation.

## Ethics Statement

All patients provided written informed consent. This study was approved by the ethical committee of the Hospitals of Lyon and by the Ministry of Research (reference number: AC-2010-1164).

## Author Contributions

PM and AH contributed to the conception and design of the study. MR, FM, and NN-T organized the database. FM performed the statistical analysis. MR wrote the first draft of the manuscript. All authors contributed to manuscript revision and read and approved the submitted version.

## Conflict of Interest

The authors declare that the research was conducted in the absence of any commercial or financial relationships that could be construed as a potential conflict of interest.
