# Supplementary material for: Joint Destruction Is Associated With All Types of Cardiovascular Events in French Rheumatoid Patients: A Real-Life Study With Very Long Follow-Up
Source: Front Med (Lausanne). 2020 Oct 30;7:556086. doi: 10.3389/fmed.2020.556086 (PMC7661545; doi:10.3389/fmed.2020.556086)
Supplement: Supplementary file 1 [file Table_1.DOCX]

Supplementary Material

# Supplementary Table

| Population description (N=571 patients) | | | |
| --- | --- | --- | --- |
| Variable | CVE+ patients (n=173) | CVE- patients (n=398) | Missing data |
| Global characteristics |  |  |  |
| Male sex - no. (%) | 68 (39.3%) | 84 (21.1%) |  |
| Year of birth | 1939 ± 11.4 | 1950 ± 12.8 |  |
| Age at diagnosis – years | 50.5 ± 14.2 | 45.8 ± 13.8 |  |
| BMI - kg/m^2^ | 26.4 ± 5.3 | 25.5 ± 5.2 | 48 (8.4%) |
| CVE - no. (%) | 173 (100%) |  |  |
| Myocardial infarction | 50 (28.9%) |  |  |
| Stroke | 28 (16.2%) |  |  |
| Acute limb ischemia | 23 (13.3%) |  |  |
| Chronic heart failure | 14 (8.1%) |  |  |
| Cardiomyopathy | 25 (14.5%) |  |  |
| Deep vein thrombosis | 27 (15.6%) |  |  |
| Pulmonary embolism | 6 (3.5%) |  |  |
| Death - no. (%) | 49 (29.3%) | 11 (2.8%) | 38 (6.7%) |
| Myocardial infarction | 15 (30.6%) | 0 |  |
| Stroke | 6 (12.2%) | 0 |  |
| Other CVE | 7 (14.3%) | 0 |  |
| Infection | 13 (26.5%) | 2 (18.2%) |  |
| Cancer | 4 (8.2%) | 6 (54.5%) |  |
| Other | 4 (8.2%) | 2 (18.2%) |  |
| Missing data | 0 | 1 |  |
| Length of follow-up – years | 19.6 ± 11.4 | 14.6 ± 8.8 |  |
| RA characteristics |  |  |  |
| ACPA positivity – no. (%) | 91 (75.2%) | 209 (56.6%) | 81 (14.2%) |
| RF positivity – no. (%) | 112 (74.2%) | 222 (57.5%) | 34 (6.0%) |
| Larsen score | 2.9 ± 1.6 | 1.8 ± 1.6 | 8 (1.4%) |
| Larsen score <2 – no. (%) | 37 (22.0%) | 199 (50.4%) |  |
| Larsen score ≥2 – no. (%) | 131 (78.0%) | 196 (49.6%) |  |
| Median DAS-28 | 3.8 ± 1.2 | 3.4 ± 1.1 | 143 (25.0%) |
| ESR – millimetres/hour | 38.9 ± 27.1 | 28.4 ± 21.6 | 51 (8.9%) |
| CV risk factors |  |  |  |
| Never smoked - no. (%) | 73 (47.4%) | 257 (65.2%) | 23 (4.0%) |
| High blood pressure - no. (%) | 116 (75.8%) | 127 (32.8%) | 31 (5.4%) |
| Diabetes - no. (%) | 28 (19.2%) | 32 (8.2%) | 37 (6.5%) |
| Dyslipidaemia - no. (%) | 73 (54.5%) | 103 (27.2%) | 59 (10.3%) |
| Treatments |  |  |  |
| NSAIDs - no. (%) | 97 (67.8%) | 249 (87.1%) | 49 (8.6%) |
| Steroids - no. (%) | 149 (86.1%) | 277 (70.3%) | 4 (0.70%) |
| Methotrexate - no. (%) | 161 (94.7%) | 377 (95.0%) | 4 (0.70%) |
| Biologics - no. (%) | 77 (44.5%) | 207 (52.0%) |  |
| Immunosuppressive agents - no. (%) | 39 (22.5%) | 37 (9.3%) |  |
| DMARDs - no. (%) | 91 (52.6%) | 176 (44.2%) |  |

**Supplementary Table 1 :** **Population characteristics at the time of analysis according to CVE status.**

If a patient had more than one cardiovascular event, the first one was used for the analysis. Quantitative variables are reported as mean ± standard deviation. RA: rheumatoid arthritis, CV: cardiovascular, CVE: CV event(s), BMI: body mass index, ACPA: anti-citrullinated protein antibodies, RF: rheumatoid factor, DAS-28: disease activity score-28, ESR: erythrocyte sedimentation rate, NSAIDs: nonsteroidal anti-inflammatory drugs, DMARDs: disease-modifying anti-rheumatic drugs.
